# Data set for diffusion coefficients of alloying elements in dilute Mg alloys from first-principles

**DOI:** 10.1016/j.dib.2015.10.024

**Published:** 2015-11-01

**Authors:** Bi-Cheng Zhou, Shun-Li Shang, Yi Wang, Zi-Kui Liu

**Affiliations:** Department of Materials Science and Engineering, The Pennsylvania State University, University Park, PA 16802, USA

## Abstract

Diffusion coefficients of alloying elements in Mg are critical for the development of new Mg alloys for lightweight applications. Here we present the data set of the temperature-dependent dilute tracer diffusion coefficients for 47 substitutional alloying elements in hexagonal closed packed (hcp) Mg calculated from first-principles calculations based on density functional theory (DFT) by combining transition state theory and an 8-frequency model. Benchmark for the DFT calculations and systematic comparison with experimental diffusion data are also presented. The data set refers to “Diffusion coefficients of alloying elements in dilute Mg alloys: A comprehensive first-principles study” by Zhou et al. [1].

**Specifications Table**

**Table:** 

Subject area	*Materials science*
More specific subject area	*Magnesium alloys*
Type of data	*Tables, Figures, Excel data sheet*
How data was acquired	*Density functional theory calculations using Vienna Ab initio simulation package (VASP)*
Data format	*Analyzed*
Experimental factors	*Not applicable*
Experimental features	*Not applicable*
Data source location	*State college, PA, USA*
Data accessibility	*Data are available here with this article*

**Value of the data**

• The comprehensive database of diffusion coefficients of 47 solutes in hcp Mg can be used by alloy designers to design better cast and wrought Mg alloys.
• The calculated diffusion data can be used to develop CALPHAD-type diffusion mobility databases for multi-component Mg alloys.
• The solute diffusion data in Mg can be used as the input for the microstructure level simulations such as phase-field simulations and finite element modeling.

## 1 Computational methods

We used first-principles calculations based on DFT coupled with transition state theory and the 8-frequency model to calculate the dilute solute tracer diffusion coefficients in hcp Mg. Forty-seven substitutional alloying elements have been considered herein, namely Ag, Al, As, Au, Be, Bi, Ca, Cd, Co, Cr, Cu, Fe, Ga, Ge, Hf, Hg, In, Ir, Li, Mn, Mo, Na, Nb, Ni, Os, Pb, Pd, Pt, Re, Rh, Ru, Sb, Sc, Se, Si, Sn, Sr, Ta, Tc, Te, Ti, Tl, V, W, Y, Zn, and Zr.

First-principles calculations based on DFT were employed to calculate the free energies needed in the diffusion equations and the 8-frequency model. The finite temperature vibrational contributions to the free energies were calculated using the quasi-harmonic approximations from phonon or Debye model. The ion–electron interaction was described by the projector augmented plane-wave (PAW) method [2] and the X-C functional was described by an improved GGA of PBEsol [3], as implemented in the VASP 5.3.2 code [4]. The PAW potentials (POTCAR files) used in the present work were released by VASP on April 19, 2012. The recommended core configurations by VASP were adopted for each element in the present work. Due to the magnetic nature of V, Cr, Mn, Fe, Co, and Ni, first-principles calculations containing these elements were performed with the spin polarization approach. An energy cut-off of 350 eV was used for the plane-wave expansion of the electronic wave functions. For the complete description of the diffusion theory used in the present work and more computational details, the reader can refer to the main article [1].

## 2 Benchmark of the DFT calculations

### 2.1 Supercell size convergence test

Solute–vacancy binding energies were calculated for Zn and Y in different supercell sizes of 36 (3×3×2 conventional hcp unit cells), 64 (4×4×2), 96 (4×4×3) and 150 (5×5×3) atoms in order to test the convergence of supercell size. Δ*V*_*X*_ is the volume difference induced by placing a single solute into pure Mg, which is a quantitative measure of the atomic size of each solute. Zn and Y represent solutes with negative and positive Δ*V*_*X*_, respectively. From the test results as shown in Table 1, we can conclude that the supercell size of Zn converges at 64 atoms and the supercell size of Y converges at 96 atoms. Therefore, for elements with large Δ*V*_*X*_ (Ba, Bi, Ca, K, Pb, Sr, and Y), 96-atom supercell was used. 64-atom supercell was adopted in calculations for all the rest of the elements.

### 2.2 *K*-point convergence test

**Fig. 1:**
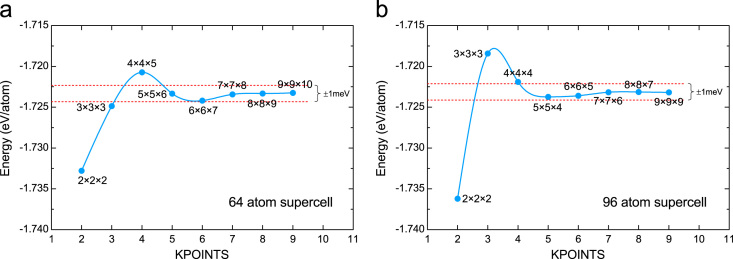
Energy convergence as a function of KPOINTS for (a) a 64 atom supercell and (b) a 96 atom supercell.

An 8×8×9 Γ-centered *k*-point mesh was used for the 64-atom supercell for the electronic integration in the Brillouin zone. For calculations using 96-atom supercells, a 5×5×4 Γ-centered *k*-point mesh was used in structural relaxation and a 7×7×7 Γ-centered *k*-point mesh in subsequent static calculations. Fig. 1 shows the energy convergence test as a function of KPOINTS in VASP for both supercells used in the calculations.

### 2.3 Thermodynamic properties of pure hcp Mg

**Fig. 2:**
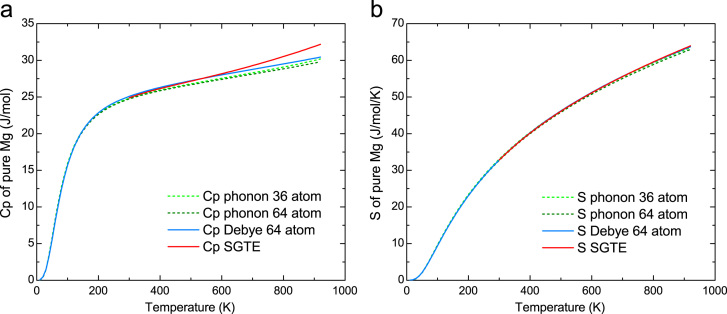
Predicted (a) heat capacity Cp and (b) entropy *S* of pure hcp Mg using Debye and phonon model in comparison with SGTE experimental data.

In order to validate the applicability of quasi-harmonic Debye model, thermodynamic properties (heat capacity Cp and entropy *S*) were predicted using both quasi-harmonic Debye and phonon model and were compared with experimental data, as shown in Fig. 2. Excellent agreement was achieved between computation and experiments.

### 2.4 Vacancy formation in pure hcp Mg

**Fig. 3:**
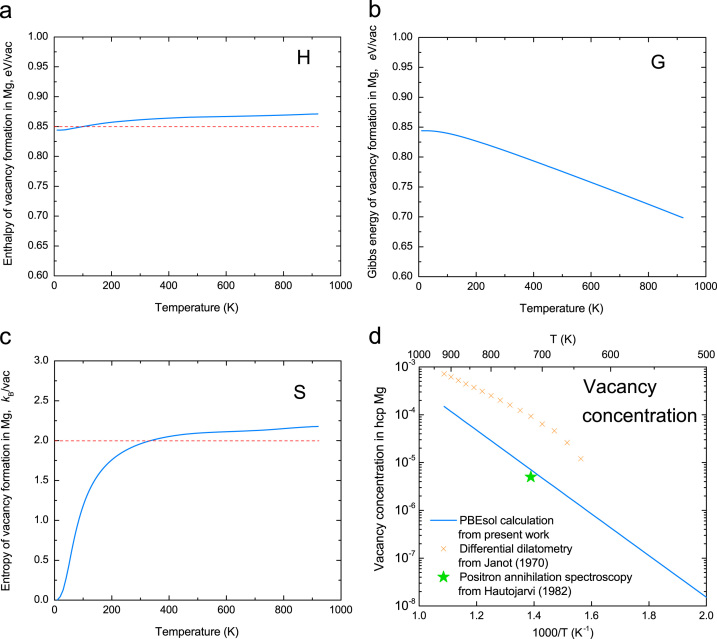
Vacancy formation (a) enthalpy, (b) free energy, (c) entropy, and (d) vacancy concentration as a function of temperature in pure hcp Mg calculated by the X-C functional of PBEsol using the quasi-harmonic Debye model. Experimental vacancy concentration data of Mg are taken from Janot et al. [5] and Hautojärvi et al. [6].

The thermodynamic properties of vacancy formation in pure hcp Mg were predicted using the quasi-harmonic Debye model and were compared with experimental data, as shown in Fig. 3.

## 3 Diffusion data

### 3.1 Plots of the calculated diffusion coefficients compared with experiments

Fig. 4, Fig. 5, Fig. 6, Fig. 7, Fig. 8, Fig. 9, Fig. 10, Fig. 11, Fig. 12, Fig. 13 show the plots of the calculated diffusion coefficients of solutes compared with available experimental data besides Al, Zn, and Sn shown in the main article [1].

### 3.2 Plots of the calculated diffusion coefficients with strong correlation effects

**Fig. 14:**
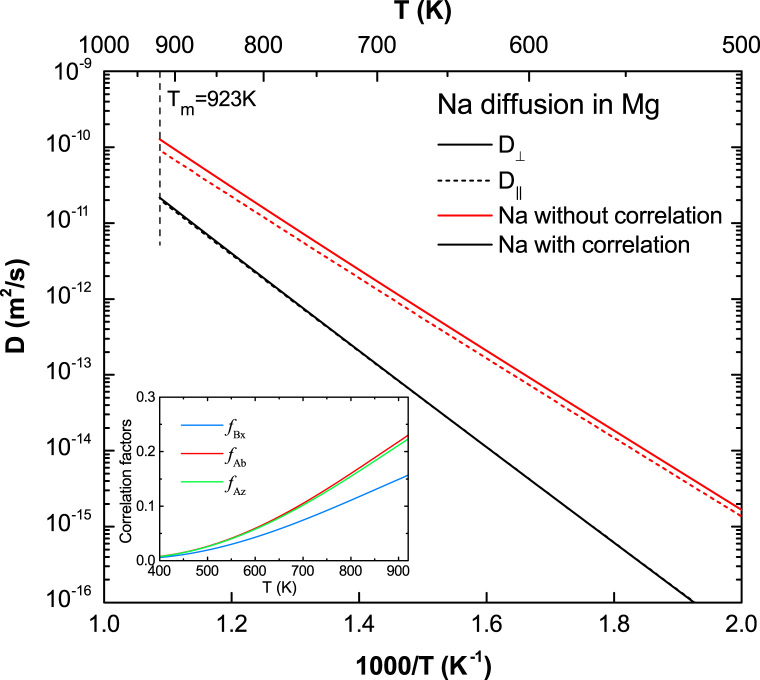
Predicted Na diffusion coefficients in Mg with and without correlation effects considered, together with the calculated correlation factors $f_{Bx}$, $f_{Ab}$ , and $f_{Az}$.

**Fig. 15:**
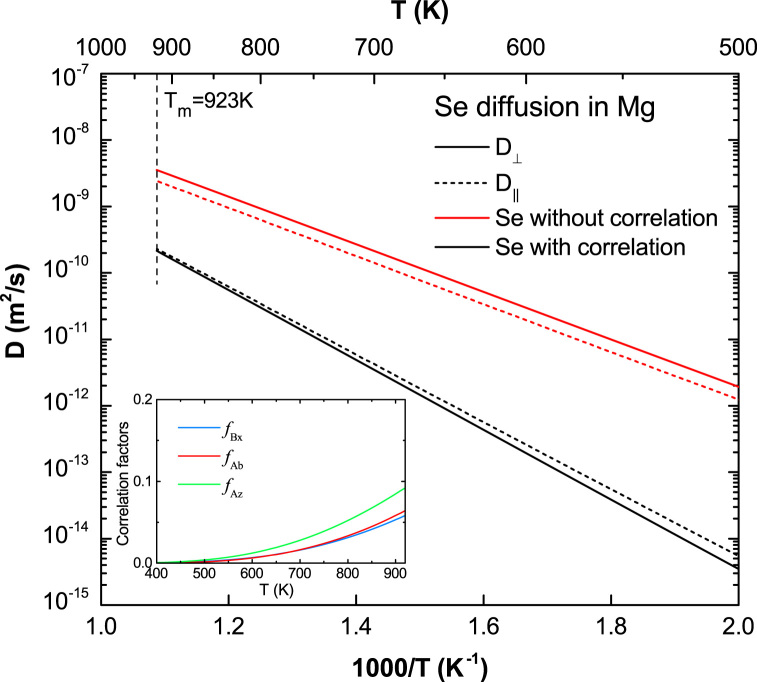
Predicted Se diffusion coefficients in Mg with and without correlation effects considered, together with the calculated correlation factors $f_{Bx}$, $f_{Ab}$, and $f_{Az}$.

**Fig. 16:**
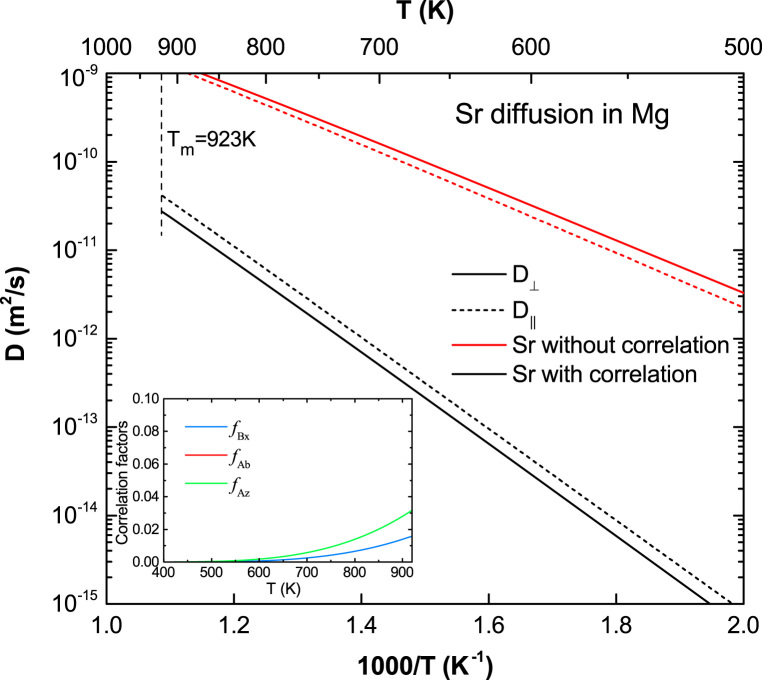
Predicted Sr diffusion coefficients in Mg with and without correlation effects considered, together with the calculated correlation factors $f_{Bx}$, $f_{Ab}$, and $f_{Az}$.

**Fig. 17:**
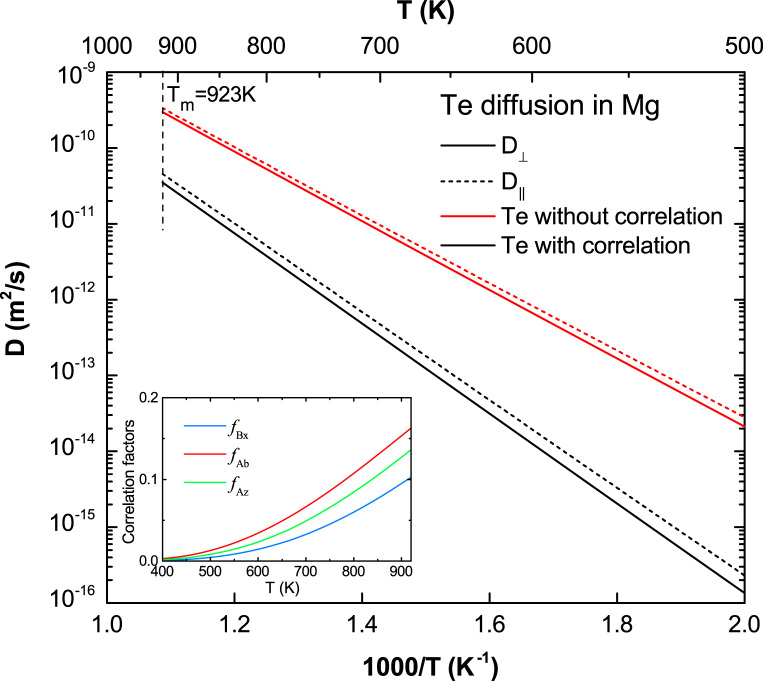
Predicted Te diffusion coefficients in Mg with and without correlation effects considered, together with the calculated correlation factors $f_{Bx}$, $f_{Ab}$, and $f_{Az}$.

**Fig. 18:**
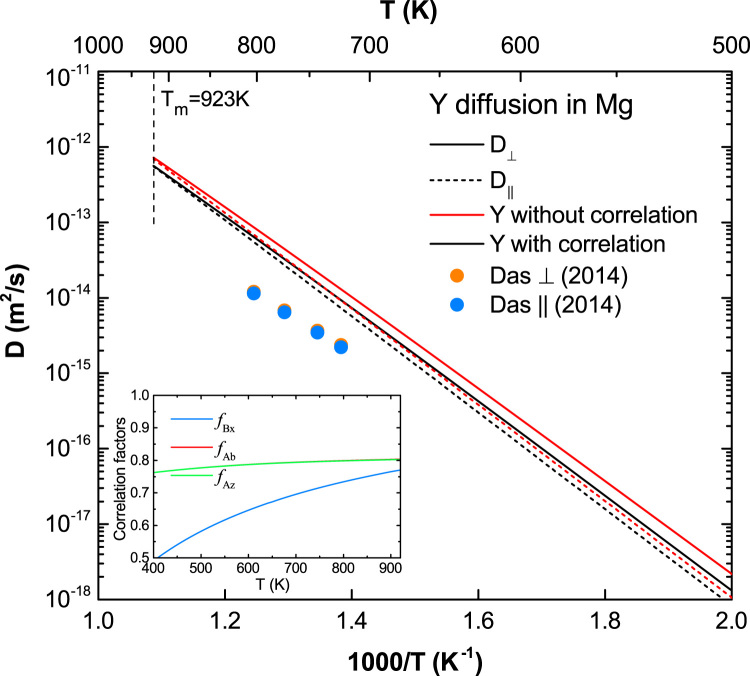
Predicted Y diffusion coefficients in Mg with and without correlation effects considered, together with the calculated correlation factors $f_{Bx}$, $f_{Ab}$, and $f_{Az}$. The experimental data are taken from Das et al. [13]. Note that $f_{Ab}$ and $f_{Az}$ almost overlap with each other.

Fig. 14, Fig. 15, Fig. 16, Fig. 17, Fig. 18 show the plots of the calculated diffusion coefficients with strong correlation effects, i.e. diffusion coefficients of Na, Se, Sr, Te, and Y in Mg (Ca in Mg in the main article [1]).

### 3.3 Diffusion data file

All the diffusion plots shown in the present work were plotted using the calculated data directly from first-principles, not the fitted Arrhenius equation. The original calculated diffusion data sets and other physical properties for each element can be found in the Excel worksheet file in the Supplementary data associated with this article.

In the diffusion data Excel worksheet file , there are the following six parts:

(1) D_basal (D_⊥_): a plot of all the predicted basal impurity diffusion coefficients in Mg for each solute.
(2) D_normal (D_||_): a plot of all the predicted normal impurity diffusion coefficients in Mg for each solute.
(3) Ratio: a plot of the ratio of basal diffusion coefficients over normal diffusion coefficients for each solute.
(4) Diffusion data from DFT: the original calculated diffusion data sets for each solute
(5) Properties from DFT: the original calculated diffusion related physical properties:
  a. Δ*V_X_*: the volume difference induced by placing a single solute into pure Mg
  b. *B*: the bulk modulus of the Mg63X dilute alloys (Mg95X for Ba, Bi, Ca, K, Pb, Sr, and Y)
  c. Ebind_basal: the solute–vacancy binding energies of solute and vacancy on the same basal plane of hcp Mg
  d. Ebind_normal: the solute–vacancy binding energies of solute and vacancy between adjacent basal planes of hcp Mg
  e. Ex: the solute migration barriers for solute–vacancy exchange within the basal plane
  f. Ex׳: the solute migration barriers for solute–vacancy exchange between adjacent basal planes
  g. Emix: the dilute mixing energy given in units of eV per atom of solute
  h. D_0__basal (*D*_0⊥_): the fitted diffusion pre-factors for the diffusion component perpendicular to the *c* axis
  i.  *Q*_basal (*Q*_⊥_): the fitted diffusion activation energies for the diffusion component perpendicular to the *c* axis
  j. D_0__normal (*D*_0||_): the fitted diffusion pre-factors for the diffusion component parallel to the *c* axis
  k. *Q*_normal (*Q*_||_): the fitted diffusion activation energies for the diffusion component parallel to the *c* axis
(6) *E*–*V* fitting results: the equilibrium properties of Mg_63_X (Mg_95_X for Ba, Bi, Ca, K, Pb, Sr, and Y): volume (*V*_0_), energy (*E*_0_), bulk modulus (*B*_0_) and its pressure derivative (*B*_0_′) for use in the Debye–Grüneisen model were obtained from an energy vs. volume equation of state (EOS) calculated from first-principles using the equilibrium volume at 0 K and at least five additional volumes (0.96, 0.98, 1.02, 1.04, and 1.06 with respect to *V*_0_).

## Figures and Tables

**Fig. 4:**
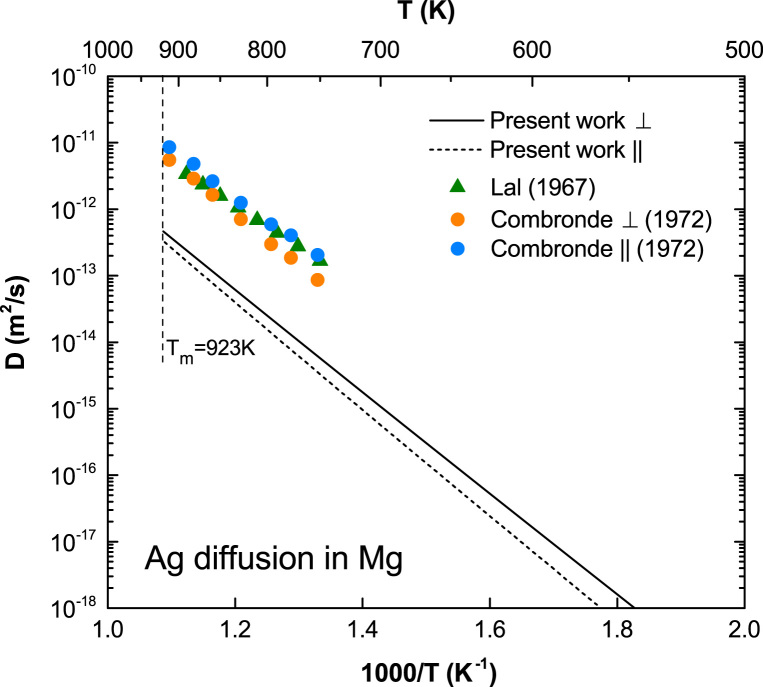
Predicted Ag diffusion coefficients in Mg with available experimental data taken from Lal [7] and Combronde and Brebec [8].

**Fig. 5:**
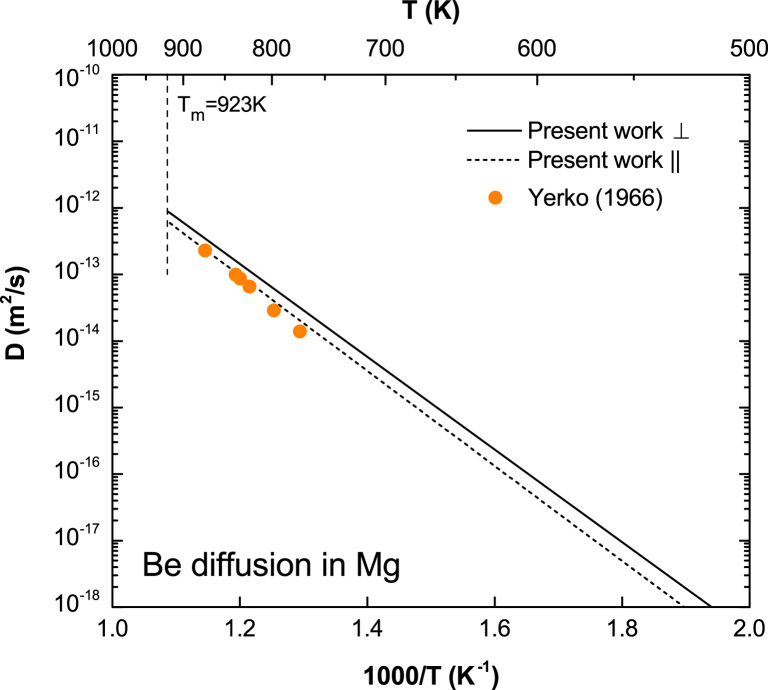
Predicted Be diffusion coefficients in Mg with available experimental data taken from Yerko et al. [9].

**Fig. 6:**
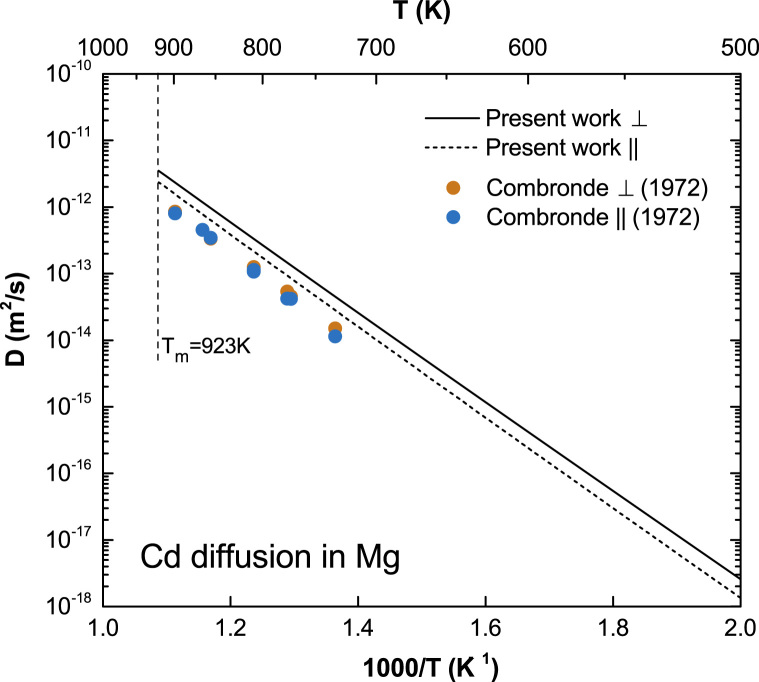
Predicted Cd diffusion coefficients in Mg with available experimental data taken from Combronde and Brebec [8].

**Fig. 7:**
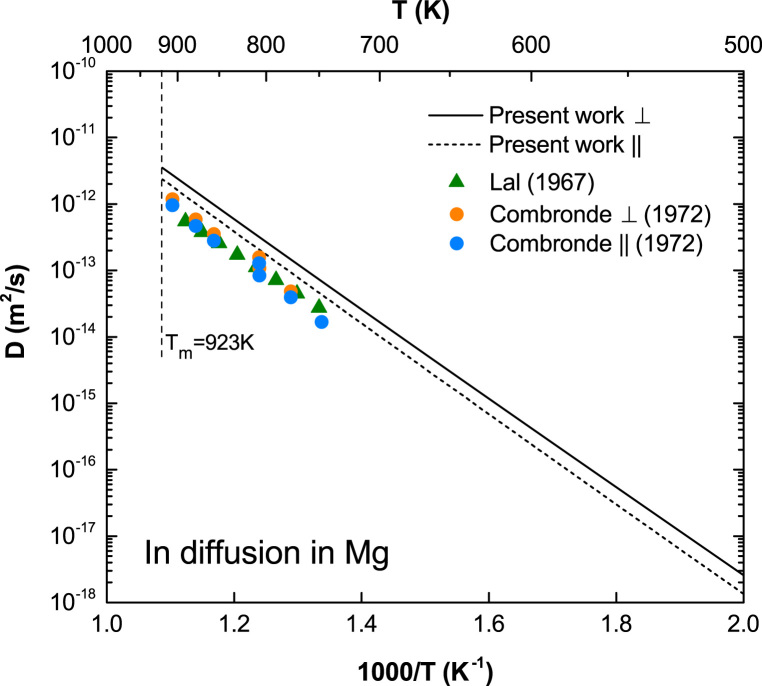
Predicted in diffusion coefficients in Mg with available experimental data taken from Lal [7] and Combronde and Brebec [8].

**Fig. 8:**
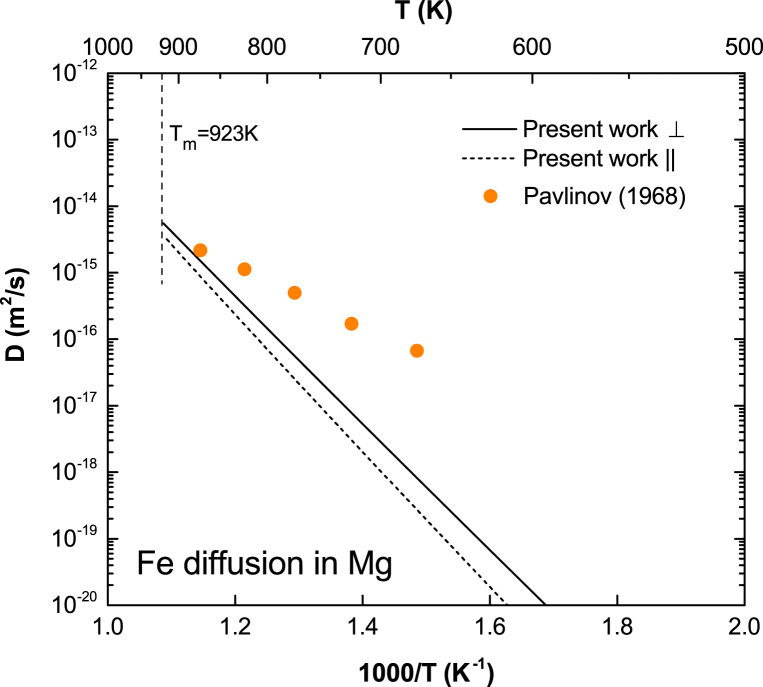
Predicted Fe diffusion coefficients in Mg with available experimental data taken from Pavlinov et al. [10].

**Fig. 9:**
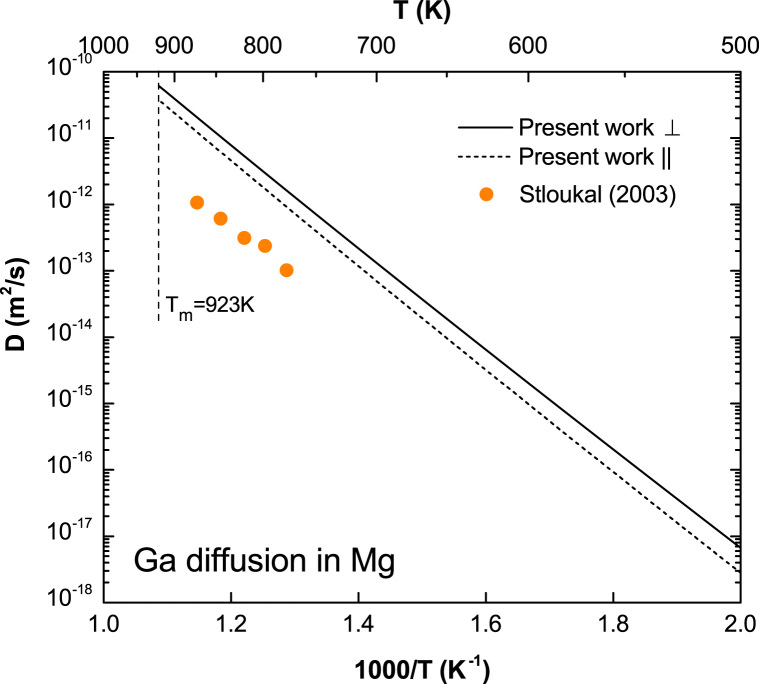
Predicted Ga diffusion coefficients in Mg with available experimental data taken from Stloukal and Čermák [11].

**Fig. 10:**
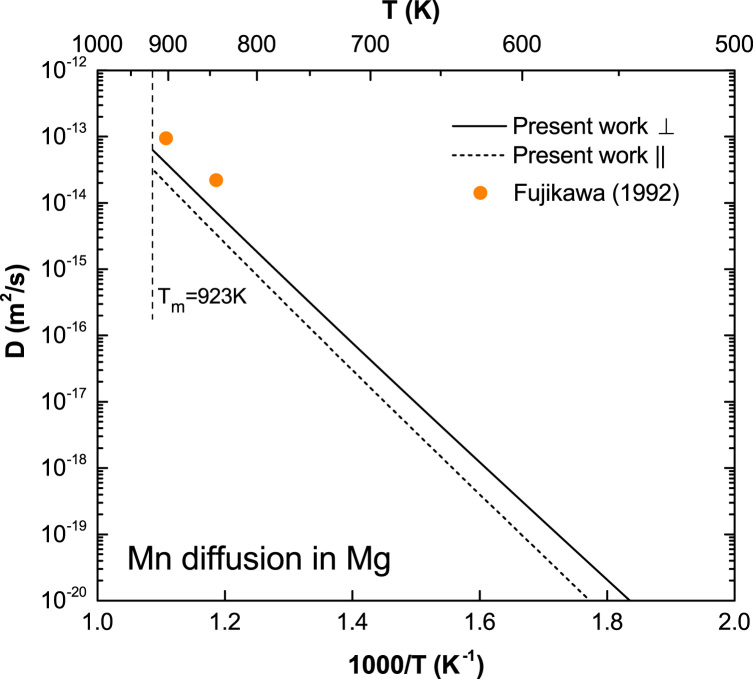
Predicted Mn diffusion coefficients in Mg with available experimental data taken from Fujikawa [12].

**Fig. 11:**
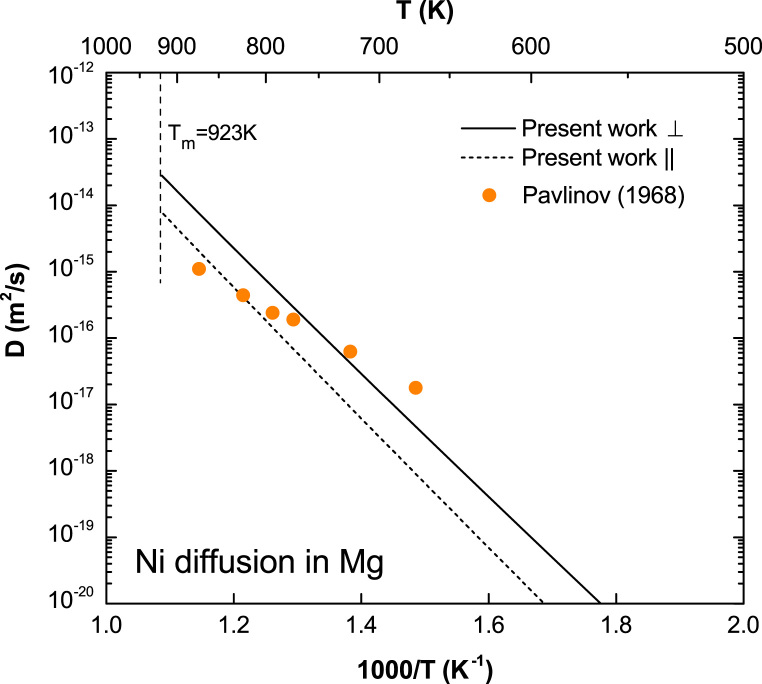
Predicted Ni diffusion coefficients in Mg with available experimental data taken from Pavlinov et al. [10].

**Fig. 12:**
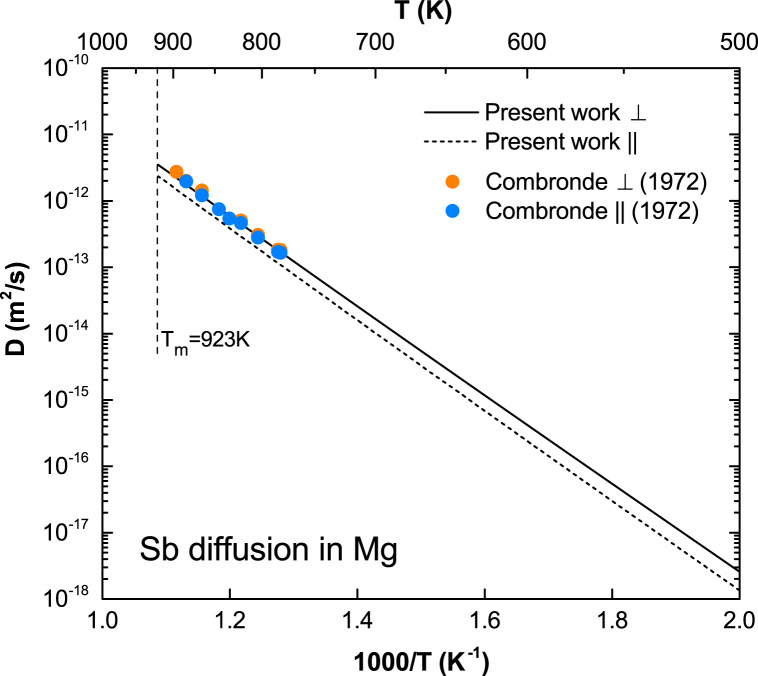
Predicted Sb diffusion coefficients in Mg with available experimental data taken from Combronde and Brebec [8].

**Fig. 13:**
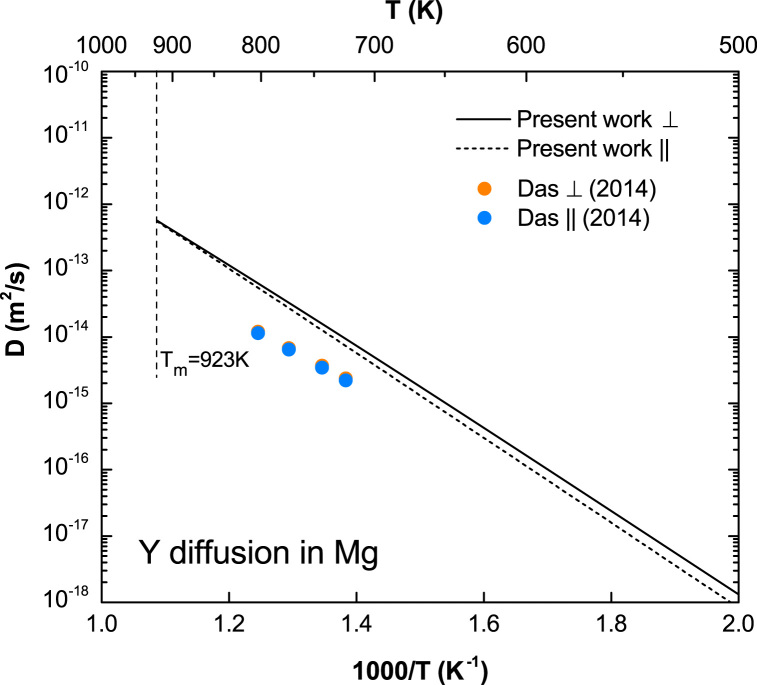
Predicted Y diffusion coefficients in Mg with available experimental data taken from Das et al. [13].

**Table 1:** Supercell size convergence of basal and normal solute–vacancy binding energies for Zn and Y. $E_{bind}^{basal}$and $E_{bind}^{normal}$ are the solute–vacancy binding energies of solute and vacancy on the same basal plane and between adjacent basal planes of hcp Mg, respectively.

Solute		Solute–vacancy binding energy (eV)			
36 atoms	64 atoms	96 atoms	150 atoms
Zn	$E_{bind}^{basal}$	0.065	0.054	0.055	
	$E_{bind}^{normal}$	0.047	0.038	0.039	
Y	$E_{bind}^{basal}$	−0.112	−0.081	−0.051	−0.051
	$E_{bind}^{normal}$	−0.096	−0.065	−0.055	−0.045

*Note:* the solute–vacancy binding energies listed here were obtained from full structural relaxations without static calculations. For accurate values of solute–vacancy binding energies in Mg, the reader should refer to Table 2 in the main article [1].
